# Depression Beyond the 6‐Month Postpartum Period: A Mixed‐Method Study Among Women in Nepal

**DOI:** 10.1111/jan.70601

**Published:** 2026-04-08

**Authors:** Sangita Pudasainee‐Kapri, Tumla Shrestha, Mary Wunnenberg, Kul Kapri, Apsara Pandey, Manisha Chapagai, Ram Hari Chapagain, Pradeep Gupta

**Affiliations:** ^1^ School of Nursing‐Camden, The Rutgers State University of New Jersey Camden New Jersey USA; ^2^ Institute of Medicine, Maharajgunj Nursing Campus Kathmandu Nepal; ^3^ Department of Political Science and Economics Rowan University Glassboro NJ USA; ^4^ Department of Psychiatry and Mental Health Tribhuvan University, Institute of Medicine Kathmandu Nepal; ^5^ Kanti Children's Hospital, Government of Nepal Kathmandu Nepal; ^6^ Department of Pediatrics Institute of Medicine, Tribhuvan University Kathmandu Nepal

**Keywords:** clinical factors, infants, mental health, postpartum depression, screening, social factors, women

## Abstract

**Aim:**

This study aimed to comprehensively examine social and clinical factors contributing to postpartum depression among women in Nepal and identify their perspectives/experiences regarding postpartum mental health.

**Design and Methods:**

The explanatory sequential mixed‐method study was conducted in two phases. In the quantitative phase, data were collected via survey of 200 postpartum women at immunisation clinics of two hospitals in Kathmandu Nepal. Measures included sociodemographic and perinatal factors and the Edinburgh Postpartum Depression Scale. Regression models were conducted to analyse data. In the qualitative phase, in‐depth interviews were conducted with 15 participants having depression scores ≥ 10. Data were analysed using the thematic content analysis method. Qualitative and quantitative findings were integrated using the joint display and the narrative weaving approach to generate results.

**Results:**

Findings show that 28% of postpartum women screened positive for depressive symptoms, requiring referral for further evaluation. Integrated findings indicate that prenatal mental health problems, low birth weight or medical conditions among infants, intimate partner violence, partner substance abuse, and a lack of postpartum social support were major contributors to postpartum depression.

**Conclusion:**

Depression is highly prevalent among women 6–12 months postpartum in Nepal and is associated with several clinical and social factors.

**Implications:**

These findings have important implications, emphasising the need for routine screening using validated tools for early identification, timely treatment and referral of women at‐risk for postpartum depression. Evidence‐based clinical and community initiatives are needed to promote positive outcomes for women and their families.

**Patient or Public Contribution:**

External experts were consulted for survey questions and qualitative interview guide, then refined those instruments based on their feedback to improve construct validity. The immunisation clinic staff provided input during study design, for participant recruitment and data collection. Key findings were presented to stakeholders to validate the interpretation of findings.

## 1 Introduction

Postpartum depression (PPD) is a significant public health concern worldwide due to its impact on not only mothers but also their infants and families. It has been identified as the most common mental health issue affecting postpartum women globally (Amer et al. 2024; Singh et al. 2021) and is a leading cause of maternal morbidity and mortality (Amiri and Behnezhad 2021; Subba et al. 2024). It is estimated to affect one in seven mothers globally (American Psychological Association [APA] 2022), with over 50% of those affected being untreated (Wang et al. 2021). The prevalence of PPD is reported to be even higher in lower and middle‐income countries (LMICs) (Carlson et al. 2025; Roddy Mitchell et al. 2023; Wasti et al. 2024). According to the World Health Organization (WHO) (2024), approximately 20% of childbearing women in developing countries such as Nepal experience PPD, which is significantly higher than those from other countries. Due to the lack of routine screening practices for mental health problems, national representative data on the prevalence of PPD in Nepal is lacking. However, recent community and hospital studies report the prevalence of PPD in Nepal between 9.4% and 41.5% (Dielemans et al. 2024; Pudasainee‐Kapri et al. 2024; Singh et al. 2021; Subba et al. 2024). Since PPD is a significant public health concern globally affecting the health and well‐being of women and children, investigation of this issue among women of childbearing age has significant international relevance.

### 1.1 PPD Diagnostic Criteria

PPD is described in the *Diagnostic and Statistical Manual of Mental Disorders, Fifth Edition‐text revision*, as a subtype of Major Depression, with peripartum onset (APA 2022). Symptoms of PPD include a depressed or low mood negatively impacting a woman's appetite and sleep, anergia, avolition, somatic complaints, feelings of hopelessness and guilt, anhedonia, and suicidal ideations and behaviours (APA 2022). Some women also experience thoughts of harming their infant and other children (National Institute of Mental Health [NIMH] 2023). These symptoms can occur during pregnancy or anytime within the first year postpartum (Radoš et al. 2024; Subba et al. 2024). While it was originally thought that PPD primarily occurred within the first 3 months postpartum, current research has shown that its onset can occur throughout the first 12 months postpartum. In a recent study, Pudasainee‐Kapri et al. (2025) found that 9.4% of Nepalese mothers 1–6 months postpartum reported higher levels of depressive symptoms needing a referral for mental health evaluation. Wang et al. (2021) reported on pooled data from several studies indicating a 17.7% global prevalence of PPD within the first 3 months postpartum, 15.9% 3 to 6 months postpartum, and 18.19% 6 to 12 months postpartum. Such research supports the need for ongoing screening for mental health issues such as PPD and anxiety throughout the entire 12‐month postpartum period.

### 1.2 Contributing Factors of Postpartum Depression

Although the exact aetiology is unknown, several factors have been associated with the onset of PPD. This includes biologic, hormonal, obstetric and social factors. Women with a prior or family history of depression are at greater risk for developing PPD (Saharoy et al. 2023). Findings from a recent systematic review of 56 studies suggest that the presence of different inflammatory markers may be associated with symptoms of peripartum depression (Silva‐Fernandes et al. 2024). Hormonal changes during the prenatal period and following delivery have also been identified as potential contributing factors to the development of PPD (NIMH 2023). Obstetric and neonatal contributing factors include pregnancy complications, issues with the infant's health (Amer et al. 2024), such as low birth weight (Pudasainee‐Kapri et al. 2025), and in some geographic regions, female gender of the infant (Subba et al. 2024; Wasti et al. 2024).

Socioeconomic factors have also been associated with a higher incidence of PPD. A lack of social support has been identified as a contributing factor for PPD (Neupane et al. 2024; Pudasainee‐Kapri et al. 2025) as social support has been found to mitigate the negative effects of stress (Liu et al. 2021; Saharoy et al. 2023). An inverse relationship between lower socioeconomic status and increased symptoms of PPD has also been identified (Singh et al. 2021), as well as fathers' lower level of education (Dawadi et al. 2020). Other socioeconomic factors related to PPD include a history of intimate partner violence (IPV), partner substance use (Dawadi et al. 2020; Wasti et al. 2024), lower education, lack of social support and financial problems (Wang et al. 2021) and the stressors of caring for an infant (NIMH 2023). Although non‐significant effects, the prevalence of PPD among women were higher based on the sex of infant (male vs. female, 17.97% vs. 20.77%) and maternal age (adolescent vs. adult, 23.34% vs. 20.35) (Wang et al. 2021). Lastly, stigma and cultural factors related to mental health issues may deter women from discussing depressive symptoms and seeking appropriate treatment (Amer et al. 2024; Wasti et al. 2024).

### 1.3 Impact of Postpartum Depression

The short‐ and long‐term effects of PPD are widespread, impacting women, their infants and families. PPD can negatively impact an infant's growth and development as it can impair mother‐infant bonding, which is crucial for positive infant outcomes (WHO 2024). Several studies have demonstrated the association between perinatal depression and mother‐infant bonding (Liu et al. 2021; Pudasainee‐Kapri et al. 2024). KC et al. (2024) reported more neurodevelopmental delays in infants whose mothers had persistent depression within the first 3 months postpartum, compared to those who did not (48.6% vs. 5.1%; *p* < 0.001). Women with depressive symptoms have also been found to have issues with sleep and difficulty feeding their infants (Dadi et al. 2020; Saharoy et al. 2023). The effects of PPD can have a long‐lasting impact, with approximately 30% of infants of mothers with PPD having behavioural issues that persist in childhood and adolescence (Dielemans et al. 2024). Despite the prevalence and significant impact of PPD, there are limited studies exploring factors contributing to PPD among women in Nepal. The empirical study was needed to examine the factors contributing to postpartum mental health outcomes among Nepali women during 6–12 months postpartum. This timeframe is particularly critical, as many women may receive substantial social support during the initial phase of childbirth but may experience a lack of similar support during the later postpartum period along with cultural and social expectations with women. Additionally, screening and identification of contributing factors of PPD beyond the initial period is crucial as women may have higher risks of developing PPD symptoms throughout the first year, with some women carrying chronic depressive symptoms with its long‐term impact on infants, women and their family.

### 1.4 Present Study

The main purpose of this study was to examine factors influencing postpartum mental health outcomes, specifically PPD among Nepalese women 6–12 months postpartum. This study also explored women's perceived experiences related to factors contributing to their postpartum mental health outcomes. To address gaps in the literature, this project investigates: (1) What is the prevalence of PPD among women 6–12 months postpartum in Nepal (2) What are the contributing factors of PPD among women 6–12 months postpartum? (3) What are the lived experiences of women regarding their postpartum mental health/PPD outcomes?

## 2 Materials and Methods

### 2.1 Study Design

The study adopted an explanatory, sequential, mixed‐method research design to understand the complex phenomena of PPD among women in Nepal. The first phase of the study included quantitative data collection and analysis. Based on the quantitative findings, qualitative sampling, data collection and analyses were done. Finally, qualitative findings were merged to expand quantitative results (Figure 1). Hence, the design included two points of integration: connecting quantitative findings for qualitative sampling and linking major quantitative and qualitative findings (Draucker et al. 2020; Fetters et al. 2013).

**FIGURE 1:**
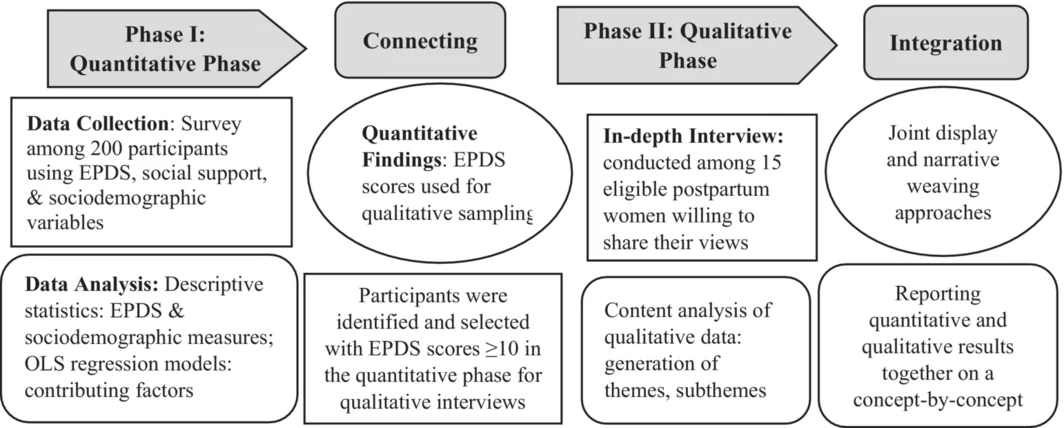
Explanatory sequential mixed‐method design. EPDS, Edinburgh Postnatal Depression Scale.

### 2.2 Quantitative Methods

#### 2.2.1 Research Sites and Participants

The study was conducted in immunisation clinics of two hospitals in Kathmandu, Nepal: Tribhuvan University Teaching Hospital (TUTH) and Kanti Children's Hospital (KCH). Two hundred postpartum women having low birth weight (LBW: infants weighing < 2500 g during birth) (*n* = 74) and normal birth weight (NBW: birth weight 2500 g or more, *n* = 126) infants were enrolled in the study. Women attending well‐baby clinics for routine vaccination of their infants were enrolled in the study using convenience sampling techniques. The inclusion criteria for participants include postpartum women who (1) are age 19 to 49, (2) have an infant six to 12 months old, (3) utilise one of the immunisation clinics of the research sites, (4) had a singleton birth with this pregnancy, (5) can report the infant's birth weight verbally or via the immunisation card, (6) is willing and able to provide written informed consent and (7) is able to read and write Nepali.

#### 2.2.2 Measures

##### 2.2.2.1 Sociodemographic and Perinatal Factors

Data on sociodemographic variables included participants' age, ethnicity, educational level and income sufficiency for their household. Infant characteristics data included age, birth weight and infants' gestational age/preterm status. Data on perinatal risk factors included maternal history of anxious and depressive thoughts during pregnancy. In addition, the history of prior LBW and/or preterm births and delivery types were recorded.

##### 2.2.2.2 Postpartum Social Support

The Maternity Social Support Scale (MSSS) was used to measure mothers' perceived social support from family, husbands/partners and friends during the postpartum period (Webster et al. 2000). It is a publicly available six‐item questionnaire using a five‐point Likert scale (1 = never, 5 = always), with higher scores indicating greater social support (Webster et al. 2000, 2011). Sample items include ‘I feel controlled by my partner’ and ‘my family is always there for me’. The MSSS has shown high reliability across prior studies, with Cronbach's alpha ranging from 0.82 to 0.90 (Mohammad et al. 2021; Webster et al. 2000).

##### 2.2.2.3 Postpartum Depression

The Edinburgh Postnatal Depression Scale (EPDS) was used to screen for depressive symptoms during the postpartum period (Cox et al. 1987). This 10‐item questionnaire (Cronbach Alpha [α] = 0.85) assesses PPD symptoms over the past 7 days, with scores ranging from 0 to 30. Sample items include ‘I have been able to laugh and see the funny side of things’ and ‘I have blamed myself unnecessarily when things went wrong’. Participants rated their feelings on a 4‐point scale, with scores of 10 or higher indicating the need for referral and further evaluation by the mental health provider (Cox et al. 1987). Items were averaged to create a composite scale in which higher scores represent greater PPD symptoms. This study used the validated Nepali version of the EDPS for screening PPD among mothers in Nepal (Bhusal et al. 2016). The EPDS has demonstrated strong validity and reliability, with Cronbach's alpha ranging from 0.74 to 0.87 in prior studies (Bhusal et al. 2016; Cox et al. 1987; Pudasainee‐Kapri et al. 2025).

##### 2.2.2.4 Control Variables

The models controlled for key perinatal and sociodemographic variables, including infant sex (male/female), participants' age (in years) and prenatal depressive thoughts (yes/no). In addition, sufficient income for households (i.e., less than a year vs. sufficient for 1 year or more) was controlled to avoid erroneous associations among key study variables in the regression model.

#### 2.2.3 Data Collection Procedures

After obtaining necessary approvals, data collection began in August 2023 and ended in January 2024. Research assistants identified eligible participants and obtained written informed consent. Then, data were collected from participants in a private area at the immunisation clinic using a paper‐based survey. The survey was first developed in English, then translated into Nepali by language and content experts. Recommendations for cross‐cultural studies were followed (Brislin 1970) and standardised and validated tools were used to measure sociodemographic characteristics, perinatal factors and depressive symptomatology.

#### 2.2.4 Data Analysis

The quantitative data collected were analysed using STATA version 17.0. Descriptive statistics were calculated for all demographic and control variables to determine participants' characteristics. Ordinary least squares regression models were conducted in STATA to analyse research questions for the effect of independent variables on the dependent variable (PPD), adjusting for all the covariates. To assess the validity of the OLS regression results, several tests were conducted to examine the key assumptions of the OLS regression model, including linearity, homoscedasticity, normality and multicollinearity. Among those, the homoscedasticity assumption was violated. To address this issue, robust standard errors were employed in the study, as they provide accurate and valid statistical inference even when the assumption of homoscedasticity (constant error variance) is not satisfied.

### 2.3 Qualitative Methods

#### 2.3.1 Participants Selection and Recruitment

Postpartum women with an EPDS score ≥ 10 identified in the quantitative phase were invited to participate and simultaneously recruited for the qualitative interviews (*n* = 15). Postpartum women who met the eligibility criteria, were willing to share their experience, and provided written consent for both participation and audio recording were purposively recruited for the study.

#### 2.3.2 Instrument and Data Collection Procedure

A semi‐structured interview guide including open‐ended questions was developed for the qualitative interview. Participants' sociodemographic information obtained in the quantitative phase were linked with qualitative data using a de‐identified identification number. To enhance the trustworthiness of the instrument, the instrument was reviewed by subject matter experts and qualitative research experts. The English version of the instrument was translated into the Nepali language, and pilot interviews were conducted with two participants, which led to minor revisions to the interview guide. Individual in‐depth interviews (IDIs) were conducted in a private, quiet area within the clinic. All interviews were digitally audio‐recorded, and field notes were taken to capture verbal and non‐verbal cues and contextual observations. Each interview lasted about 60 min. Recruitment ended after 15 interviews in which the sample size was determined based on data saturation as no new substantial information emerged.

#### 2.3.3 Data Analysis

Qualitative data collection and analyses were conducted simultaneously. Recorded interviews were transcribed and translated into English. The research team and research assistants reviewed interview recordings for consistency by repeatedly listening to the audio recordings, reading the transcripts and conducting cross‐validation with notes taken during the interviews. Data were analysed manually using the thematic content analysis method. The themes and subthemes were comprehensively described and contextualised with relevant quotes.

#### 2.3.4 Trustworthiness

Lincoln and Guba (1986) describe four criteria for conducting qualitative research: *credibility, dependability, transferability* and *confirmability* to ensure the rigour of the study. Credibility was upheld by expert consultations, by building trust and mutual rapport with participants, spending time in in‐depth interviews and voluntary participation in the interview to share their descriptive experiences of PPD. Field notes were maintained during the interviews. Dependability was ensured by using a consistent interview guide during in‐depth interviews and prompt translation/transcription of audio recordings. Transferability was maintained by a comprehensive research process along with direct quotes to support themes/subthemes. For confirmability, the authors comprehensively reviewed the process of data analysis and study findings. All the authors read and agreed on the final manuscript before submission.

### 2.4 Integration of Findings

Integration in mixed‐methods research involves combining qualitative and quantitative strands to generate a comprehensive understanding of the phenomenon under study. In this study, integration occurred at two distinct points (Figure 1). First, quantitative findings guided the recruitment of participants for the qualitative phase (Draucker et al. 2020; Fetters et al. 2013). The second level of integration was performed after qualitative data analysis for the interpretation and reporting of overall findings. Two approaches were used for integration of both findings: the joint display and the narrative weaving approach based on prior research (Draucker et al. 2020; Fetters et al. 2013). Using the joint display approach, qualitative and quantitative findings were presented together in a table to facilitate comparison and interpretation. In the narrative weaving approach, findings from both strands were integrated and described together on a concept‐by‐concept basis for a comprehensive understanding of the study phenomena. The integration results were interpreted as confirmation (when findings from both data strands supported each other), expansion (when findings complemented each other by providing additional insights into different aspects of the phenomenon), or discordance (when qualitative and quantitative findings were inconsistent or contradictory) (Fetters et al. 2013).

## 3 Results

### 3.1 Sociodemographic Characteristics

Descriptive statistics of participants are displayed in Table 1. The results indicate that the mean infant age was 9.96 months (SD = 1.424) and the mean age of participants was 29.02 years (SD = 4.631). Participants were ethnically diverse, with 56.5% Brahmin/Chhetri, 36.5% Janatati, 6% Dalit and 1% Madhesi. About 37% of the participants had a high school education. Approximately 53% of participants reported having caesarean delivery, and 90% gave birth in a public hospital. A significantly large proportion (90.0%) of participants were currently breastfeeding or feeding pumped breastmilk to their infants. Thirty‐nine percent of participants reported having anxious thoughts, and 29% reported having depressive thoughts during pregnancy. Fifty nine percent of participants had four or more prenatal check‐ups during their pregnancy.

**TABLE 1:** Sociodemographic characteristics of study participants (*N* = 200).

Variables	Counts & %
Participants' age: Mean = 29.02 (SD = 4.631)	
Infant age in months: Mean = 9.96 (SD = 1.424)	
Infant sex	
Female	88 (44.0%)
Male	112 (56.0%)
Participant's Ethnicity	
Dalit	12 (6%)
Janajati	73 (36.5%)
Madhesi	2 (1.0%)
Bramhan Chetri	113 (56.5%)
Type of delivery	
Vaginal	92 (46.0%)
Vaginal Assisted Delivery	2 (1.0%)
Caesarean Section	106 (53.0%)
Breastfeeding to infants	
After birth	184 (92.0%)
Currently feeding/feeding pumped milk	180 (90.0%)
Prior LBW	16 (8.0%)
Participant education level	
Primary	5 (2.5%)
Middle school	19 (9.5%)
Secondary	28 (14.0%)
Higher secondary	74 (37.0%)
Bachelor	39 (19.5%)
Master's level/higher	35 (17.5%)
Income sufficient for household expenditure	
< 6 months	41 (20.5%)
6–11 months	19 (9.5%)
12 months	75 (37.5%)
> 12 months	65 (32.5%)
Anxious thoughts during pregnancy	78 (39.0%)
Depressive thoughts during pregnancy	58 (29.0%)

Abbreviation: SD, standard deviation.

### 3.2 Integrated Findings

Findings of the quantitative study were integrated with the findings of the in‐depth qualitative study among participants during the second phase.

### 3.3 Prevalence of Depressive Symptoms

#### 3.3.1 Quantitative Findings

A significant proportion of the participants had moderate levels of depressive symptoms with EPDS scores 6–9 (20.5%; 41 out of 200 participants). 28% of participants (*n* = 56 out of 200) had EPDS scores 10 or higher, indicating the need for a mental health referral (see Table 2).

**TABLE 2:** Distribution of postpartum depression scores among participants (*N* = 200).

EPDS scores	Counts	%
Mild (0–6)	103	51.5
Moderate (7–9)	41	20.5
Severe (> 9)	56	28

*Note:* 28% of participants who had EPDS scores of 10 or higher met the criteria and received a referral for further evaluation and monitoring.

Abbreviation: EPDS, Edinburgh Postnatal Depression Scale.

#### 3.3.2 Qualitative Findings

##### 3.3.2.1 Theme 1. Mental Health Symptomatology

Qualitative exploration of mental health problems among women 6–12 months postpartum has emerged as a theme, namely ‘Mental Health Symptomatology’ based on subthemes. Findings show that depressive symptoms exist among women during the 6–12 months postpartum period. Some of the participants reported being depressed and experiencing suicidal thoughts during both pregnancy and the postpartum period. They shared their experience of multiple somatic symptoms, self‐harming behaviours, and a lack of strength and confidence. The theme is based on two different subthemes.

###### 3.3.2.1.1 Negative Emotions and Self‐Harming Behaviour

Participants shared their experiences of negative emotions such as stress and worry, fear and anxiety, feelings of regret, hopelessness, and disappointment regarding their pregnancy, delivery and care of their infant. Some of the participants reported harming self and experiencing suicidal thoughts related to negative emotions during the postpartum period.

When there is a lot of tension, I injure myself, sit and hit the wall. My mind does not work, so I do so. I cry, I cry now and then there is no one to support me and I want to do so. (P8)

It was very difficult at that time, it was really difficult at that time, I felt like I wanted to die, that was it. (P12, 207‐8, pp2)

Some participants reported self‐harm and violent behaviour to express their feelings and cope with stress, while others expressed feelings of deterioration of personal strengths. A participant shared,

When I feel angry or worried, I bother myself, and unknowingly, I feel a seizure and twisting of hands and feet. I can't express myself as he can't understand. In tension, I injured myself, sat and hit the wall. My mind does not work, so I do so. (P8)

###### 3.3.2.1.2 Physical Symptoms and Reality Unacceptance

Along with mental health symptoms, some participants reported experiencing somatic symptoms, such as headaches, eye pain and knee pain. They frequently sought medical help for diverse health problems. Although some reported being referred to a mental health provider, some participants reported not consulting the psychiatrist, despite the recommendations. A participant verbalised,

Sometimes I have trouble swallowing my throat, it feels like my throat is obstructed. Ears become dull, headache, eye pain etc. The knee was also painful, and consulted with rheumatic specialist, again nothing was found. The doctor finally advised for psychiatrist consultation. However, I didn't consult them. (P5)

### 3.4 Contributing Factors of Postpartum Depression

#### 3.4.1 Quantitative Findings

Table 3 highlighted comprehensive clinical and social factors predicting PPD 6–12 months postpartum, controlling for mothers' age, infants' sex, household income and prenatal depressive thoughts. The ordinary least squares regression model indicated that LBW was modestly associated with PPD among women 6–12 months postpartum (*β* = −1.153, SE = 0.683, *p* < 0.10). Additionally, anxious thoughts during pregnancy significantly predicted a higher risk for PPD (*β* = 2.758, SE = 0.667, *p* < 0.01).

**TABLE 3:** Ordinary least square regression model predicting PPD by clinical and social factors.

	Coefficients (*β*)	Robust SE	95% CI
Low birthweight dummy	−1.153*	0.683	[−2.499 to 0.195]
Experienced anxious thoughts in pregnancy	2.758***	0.667	[1.442 to 4.074]
Exposure to violence/victimisation	0.330*	0.196	[−0.057 to 0.718]
Postpartum social support	−0.462***	0.155	[−0.768 to −0.156]
Educational status	0.520**	0.250	[0.027 to 1.014]
Food security	−1.118***	0.316	[−1.742 to −0.494]
Income sufficient for household expenses	0.0607	0.360	[−0.649 to 0.771]
Experienced depressive thoughts in pregnancy	0.508	0.806	[−1.081 to 2.097]
Infant sex	−0.488	0.612	[−1.695 to 0.720]
Participants' age	−0.0460	0.0608	[−0.166 to 0.074]
Constant	21.49***	4.671	[12.278 to 30.706]
*N*	200		
*r* ^2^	0.401		
Root MSE	4.284		

*Note:* Β: standardised coefficients, *p < 0.10, **p < 0.05, ***p < 0.01.

Abbreviations: Dependent variable: CI, Confidence interval; PPD, Total scores of Edinburgh Postnatal Depression Scale (EPDS) for each participant; SE, Standard errors.

In terms of social determinants, results indicated that variables including social support, educational status, exposure to violence and victimisation, and food insecurity significantly predicted PPD in Nepali women 6–12 months postpartum, controlling for several factors in the model. More specifically, exposure to violence from husbands/partners or other individuals was significantly associated with PPD among women (*β* = 0.330, SE = 0.196, *p* < 0.10). Women who experienced higher levels of social support from partners/husbands, family, and/or friends during the postpartum period had significantly lower rates of PPD (*β* = −0.462, SE = 0.155, *p* < 0.05). Similarly, food insecurity was significantly associated with PPD among women (*β* = −1.118, SE = 0.316, *p* < 0.01). Interestingly, results also indicated that higher levels of education among mothers significantly predicted more depressive symptoms among Nepali women (*β* = 0.520, SE = 0.250, *p* < 0.05).

#### 3.4.2 Qualitative Findings

Qualitative exploration of women's perspectives regarding contributing factors of depression identified three thematic areas including Spouse, Family and Social Support; Socioeconomic Factors; and Clinical Factors along with eight subthemes (see Table 4).

**TABLE 4:** Joint display of contributing factors for depression beyond 6 months postpartum period among participants.

Integrated quantitative (*n* = 200) and qualitative (*n* = 15) findings	Interpretation
Postpartum Depressive Symptoms Quantitative: Edinburgh Postnatal Depression Scale (EPDS) Scores Moderate (6–9): 41 participants (20.5%) Severe (10 or higher): 56 participants (28%) Qualitative: Mental Health Symptomatology Negative emotions, self‐harming behaviour and suicidal thoughts Physical symptoms and reality unacceptance	Higher depressive scores among significant number of participants Survey findings supported by women's expression of various negative emotions, suicidal thoughts, self‐harm and somatic symptoms (Expansion)
2 Factors Influencing Postpartum Depression Quantitative: Predictors of Postpartum Depression Postpartum social support (spouse, family and friends) (*β* = −0.462, SE = 0.155, *p* < 0.05) Exposure to violence (*β* = 0.330, SE = 0.196, *p* < 0.10) Lack of food security (*β* = −1.118, SE = 0.316, *p* < 0.01) Prenatal anxiety (*β* = 2.758, SE = 0.667, *p* < 0.01) Low birth weight (*β* = −1.153, SE = 0.683, *p* < 0.10) Qualitative: Contributing factors of Postpartum Depression i. Spouse, family and social support Spouse relationship and support Family and social support Peer support ii. Socioeconomic factors Unwanted pregnancies and infant sex preference Substance use and intimate partner violence Economic hardship iii. Clinical factors Prenatal mental health problems including anxiety and depression Preterm birth, low birth weight and/or sick infant	Spouse, family and social support, socioeconomic and clinical factors as the main contributors for PPD (Confirmation/Expansion)

##### 3.4.2.1 Theme 2. Spouse, Family and Social Support

Participants who had support from their husbands, in‐laws, parents and other maternal‐side relatives seemed to experience less psychological distress such as stress and a low mood. Some participants said that they went back to work and received help from family to care for the infant/other children. However, participants who reported inadequate support from their husbands, family and friends had higher levels of depressive symptoms. The theme is based on three subthemes.

###### 3.4.2.1.1 Spouse Support and Relationship

Participants identified their relationship with their spouse and a lack of support as key influencing factors in the development of depressive symptoms. Participants living solely with their husbands expressed disappointment and misery related to inadequate support and having the sole responsibility of childcare. Participants, specifically those with a LBW and/or a sick child, reported experiencing fatigue, exhaustion and monotony from continuous caretaking of their children.

I can't even ask anyone to hold my child for a while. Once I asked my husband, but he bluntly replied that he was not there to look after the child. He says, ‘I don't have time; I cannot leave the office to others’. It is not that money is every thing, it is just support, isn't it? I have a sole responsibility to look after the child all the time. It is natural to be fed up and bored. (P. 14)

Some participants verbalised that they experienced negative behaviours from their spouse and were treated poorly during the postpartum period. This included scolding, blaming them for their child's illness, and criticising their ability to care for their child. They expressed frustration and disappointment over their husband's behaviour and responses. Additionally, patriarchal societal beliefs and social or cultural expectations further strained the relationships among couples leading to disparate feelings. A participant reported her husband saying, “You are not the only person to become a mother, women in the village deliver the child, take care of them, and work all the household chores as well, and you can do nothing.” My husband says bluntly (P6).

In contrast, participants who had a good relationship with their husband and received adequate support for childcare and other household chores reported being able to manage their responsibilities with less burden or stress.

A participant shared, 'Although my husband has pressure from his work and cannot give adequate time, he does a lot when he is at home like cooking, cleaning and other chores too. So, we are managing care of our child well and I have no psychological burden related to it.' (P15).

###### 3.4.2.1.2 Family and Social Support

Participants shared that perceived family support is receiving assistance and guidance during childbirth and for childcare, especially those from extended families (i.e., relatives beyond parents and children, encompassing grandparents, aunts, uncles and cousins, who may live together). Some participants reported receiving good family support, guidance and care following childbirth. They also reported positive family support for childcare and being involved in major decision‐making processes.

After delivery, my family helped me a lot, even my parents helped me then. Right now, I have left my child at home, they (in‐laws) are looking after him. He doesn't look for me most of the time. I have no difficulties for child rearing. (P2)

My mother in‐law supported me during delivery. As a postpartum woman, I received good care from both at home and at my maternal home. My female close relatives from both sides guide and support me for taking care of the child. Therefore, I am enjoying with parenting. (P7)

Participants residing in urban cities, and being far from extended family, faced significant challenges in seeking family support following childbirth or during sickness (self/their infant). For some participants, family support was only for a short time. Additionally, participants who lived in urban areas and away from their immediate family received less family support due to family members' work commitments and challenges in their personal lives. Some participants had to care for their infants and other children alone without their husband's help and felt helpless.

My mother‐in‐law denied coming at the time of childbirth and when we asked my sister‐in‐law, she also denied. After all it was our first child and we had no experience at all, we felt rejected and insecure about the situation. (P7)

After three months' stay, my mother returned due to family problems. After that, I did all by myself in raising the child myself. It is not because I cannot feed myself, but it is obvious to expect some affection during the postpartum period. I cried a lot, but no one from our family and maternal home showed concern and support. It was challenging and distressing for me to take care of children by a single person. (P14)

My baby needed hospitalisation, and I had no one by my side to help. I didn't get to eat on time, so I had to walk through the hospital to collect the reports by myself. That is why I had profuse bleeding and fainted and was hospitalised for a week. What to say about my misfortune? (P6)

However, A participant who went to the hometown in the rural area during the postpartum period with her LBW infant described that family and social context for childcare in rural areas was extremely stressful. The participant said, ‘we need to hear comments from families like “she came here to raise her child” “negative comments about my child”, “unnecessary gossip”, “talking back and forth”. There is less opportunity to share things with my husband as he often goes around the village with his friends and is not available at home. All these situations create tension.’ (P7).

###### 3.4.2.1.3 Peer Support

Peer support has been identified as a protective factor for postpartum women's mental health. Some participants received reassurance and guidance on caring for a sick or premature infant from their peers. They also mentioned alleviation of stress and worries, seeing other mothers facing similar issues, such as having a LBW infant. A participant verbalised,

There were 2‐3 other women who had a child with very low birth weight (birth weight < 1500 grams). The most crucial matter is that seeing others with the same experience gave me courage. I also learned care of my child with them (P5)

##### 3.4.2.2 Theme 3: Socioeconomic Factors

Socioeconomic factors including the infant's sex preference, unplanned pregnancies, IPV, exposure to substance use, and financial hardship play a crucial role in contributing to PPD among women in Nepal. Some participants reported experiencing stress and emotional problems due to unplanned pregnancies, with others facing increased pressure from their husbands regarding infant sex, leading to emotional threats. Violence was often exacerbated by spousal substance abuse, contributing to their fear, helplessness and isolation as they endured physical and emotional abuse. Additionally, financial hardship was a common theme experienced by women who were financially dependent on their husbands, contributing to increased anxiety and depressive symptoms. The interaction of these socioeconomic stressors contributed to feelings of hopelessness, low self‐esteem and emotional distress.

###### 3.4.2.2.1 Unwanted Pregnancies and Infant Sex Preference

Participants reported feeling stress and worry related to unplanned pregnancies. Some participants shared their happiness for having a child of the preferred sex and shared about the preference of their husbands. Some of them received inadequate support and emotional abuse from their husbands because of having daughters. One participant was even threatened about a possible second marriage by her husband if she didn't give birth to a son next time. Participants shared:

We had not planned to have the child, but I became pregnant unknowingly. We were not ready to share the news with anyone and were planning to terminate the pregnancy. (P12)

I didn't want to have a child, but my husband insisted on another one. I tried to abort it when I was 5 months pregnant. I desired a son and when I knew it was a son, I felt joyous. (P8)

My husband would like to terminate if it was a daughter. We checked sex of the baby near Bir Hospital by paying Rs. 3500. It was a daughter. (P14)

My husband says that he wants a son. He said, ‘I didn't ask you to have a child and that, a daughter’. If you don't give birth to a son, I will marry again. (P6)

###### 3.4.2.2.2 Substance Use and Intimate Partner Violence

Violence resulted in increased depressive symptoms among women. Some participants shared their experiences of periodic IPV during pregnancy and the postpartum period. They expressed fear of unpredictable, painful events associated with their husband's behaviour. Some participants also verbalised being victims of various forms of violence due to their husbands' substance use behaviours (such as regular drinking). The husband's abusive behaviour left women feeling dismal, helpless, and having crying spells. They also reported that they received little to no physical, emotional, or financial support in raising their child. Some participants were unable to share their experiences of IPV (drinking and torture) with their family and friends. Instead, they were living with low self‐esteem and feeling defenseless due to the lack of social support. Participants said,

I eloped with the person I fell in love with, he often beat me without any reason. I had to agree to whatever he would say, if not, he would beat me up with sticks and I used to have bruises all over my body. I could not bear it anymore and I escaped from the house when my daughter was 2 years old. But he took me back home again. He did not hit me for a while after that. Then, when I became pregnant again, the violence resumed and he began hitting me again. When I asked him a reason why he was beating or when I tried to defend him, he beats me even more and threatens to kill me by choking me and so on. (P6)

Sometimes my husband threatens that he takes my child, and he will not give my daughter back to me. Sometimes I am fearful that something will happen while he is drunk. Those situations or feelings make me cry. If I cry, he will beat even more, so I try to hide my tears and cry when I'm alone. There is no other way or alternative for me as I escaped from my parents' house and had an eloped marriage without my family's approval. So, I can't share my pain and sorrow with my family. I haven't told my parents that my husband drinks and tortures me because they would get more worried. It is very painful; the main reasons of my suffering are my husband's behaviour and my financial situation. (P6)

In addition to experiencing their partners' substance abuse, a participant shared that substance use and exposure to family violence contributed to increased mental health issues and leaving the extended family. A participant shared, ‘My father‐in‐law drinks a lot, scolds and makes arguments with everyone all the time especially with mother‐in‐law. He even misbehaves with me. We feel very bad, that's why we came here so quickly’. (P7).

###### 3.4.2.2.3 Economic Hardship

One participant shared experiencing significant levels of stress from economic hardship during her pregnancy, at childbirth, and during the postpartum period. Loss of employment during pregnancy and financial dependency on her husband resulted in limited resources to fulfil their basic needs which led to additional relationship conflicts. Some participants expressed feelings of regret, sorrow and disappointment due to financial hardship. Participants shared:

Money is a major problem. I used to work but had to stop when I was 3 months pregnant. I couldn't continue my work at that time. After losing my job, I got more dependent on my husband for money, but he refused to give me any time when I asked which led to our conflicts. I got so depressed and overwhelmed and worried about how to raise my child. I was also worried about what my future holds, especially if I cannot go abroad to earn money. My husband provides no financial support that increased my stress and worries. (P6)

I am worried about my husband. The worries increased even more after I had the child. I haven't received any financial help from my husband. (P4)

Some participants shared the financial burden caused by their child's illness, treatment costs and/or hospitalisation. The need to take the sick child to a private hospital for treatment due to unavoidable situations further exacerbates financial strain. Experiencing multiple challenges to manage expenses while relying on one person's income, added to women's stress and anxiety levels. A participant verbalised,

The second child (LBW) frequently gets sick, we need to take her to an expensive hospital for prompt management. We need to pay 16 to 17 thousands per month for house rent. In addition, we need to pay the school fee for our eldest daughter. Managing money is great trouble for us. (P14)

##### 3.4.2.3 Theme 4: Clinical Factors

Some women reported having increased mental health issues including anxious and depressive symptoms during their pregnancy. They also expressed infant health‐related stressors as contributing to increased distress during the postpartum period.

###### 3.4.2.3.1 Prenatal Mental Health Problems

Some participants reported increased mental health problems, including depressive and anxious symptoms during their pregnancy.

At first, I felt like I was going into depression. Because I was busy with going to the office, here and there. Suddenly, I felt like engaged, barrier for grow up and better without giving birth. (P13)

It was not consensual pregnancy. We have no plans to have a child. I was planning to go for the Depo‐Provera injection. He kept a relationship after drinking and I became pregnant. Later, he used to say why did you become pregnant? Why didn't you take the injection? Therefore, I was very much worried and stressed during pregnancy. (P6)

During pregnancy, I had a lot of anxiety attacks and was also depressive feeling like I wouldn't survive. For 1‐5 months, I wouldn't like talking to anyone, and eat anything, feeling like completely depressed. (P12)

###### 3.4.2.3.2 Preterm Birth, Low Birth Weight and/or Sick Infant

Participants with a sick infant or those who had a LBW or preterm infant felt additional physical and emotional burdens with childcare. Participants shared their continuous and exhaustive engagement in the care of the LBW or sick child, leading to a lack of self‐care and restrictions on personal and social activities. Women reported ongoing worry and fear related to their child's health, compromised activities/routines due to the need for follow‐up care for their child, and disappointment regarding their infant's frequent illnesses. They also verbalised their fear and insecurity related to their infant's frequent morbidities and hospitalisations as well as physical care needs and financial burdens. Participants shared,

My child was born before the term and was only 1600 grams. The situation was so stressful that there was no place for more. I was very tense about what would happen next to the child and watched frequently. I took him to the hospital regularly like weekly. I could not sleep at night. I was awake almost the whole night for three months and slept very little for up to four or five months. I am alive now with a hard struggle. (P14).

I am caring for my baby the whole day and night for his betterment. He couldn't even sleep. He grew up placing in my chest for 2‐3 months, because he was so small at birth. (P9)

I am very worried because my child is not well. He has a problem with urination due to narrowing of the urethral opening. Situation is not improving despite the application of medications at a regular basis I am applying. (P2)

When I sit to eat, my daughter cries, and the food gets cold. Afterward, I lost my appetite for food. There is no time for food. I didn't want to make her cry. I didn't have time to eat as I had to finish many things while she was sleeping. So, eating was not a priority. It was full of struggle; I wanted to cry from time to time. (P14)

## 4 Discussion

The aim of this mixed‐method study was to examine the social and clinical factors contributing to PPD among women 6–12 months postpartum in Nepal and identify their perspectives and/or lived experiences regarding their postpartum mental health. While evidence on the prevalence of PPD during the early postpartum period is available, there is limited data of PPD prevalence and its contributing factors specifically for the 6–12‐month postpartum period, particularly in Nepal. In this study, 28% of women were identified as experiencing symptoms of PPD during 6–12 months postpartum that warranted referral for further evaluation. This finding falls within the previously reported range for Nepal (9.4%–41.5%) (Dielemans et al. 2024; Pudasainee‐Kapri et al. 2024; Subba et al. 2024; Wasti et al. 2024). However, the rate of PPD is higher for women 6–12 months postpartum in Nepal than the 20% prevalence (one in five women) reported for developing countries (WHO 2024) and the pooled prevalence of 23.1% found in a meta‐analysis of PPD in low‐ and middle‐income countries (Roddy Mitchell et al. 2023). These findings are also consistent with longitudinal studies conducted in Italy and Spain, which have reported persistent prevalence of PPD throughout the first year, with the highest rates observed at 12 months postpartum (Míguez and Vázquez 2023; Molgora et al. 2022). Similarly, qualitative findings of this study revealed manifestation of depressive symptoms among women in various forms such as feeling depressed, experiencing suicidal thoughts, and engaging in self‐harming behaviours coupled with multiple somatic symptoms. These findings underscore the burden of perinatal depression throughout the first year postpartum and highlight the need for ongoing screening and preventive interventions (e.g., educational programs) for postpartum women (Míguez and Vázquez 2023; Molgora et al. 2022).

Mixed‐method findings of our study indicated several key factors contributing to depressive symptoms among women 6–12 months postpartum in Nepal. Specifically, the quantitative results indicated that several key clinical and social factors including LBW, anxious thoughts during pregnancy, lack of social support, education status, exposure to violence and victimisation with husband or others, and food insecurity were significant predictors of PPD among women 6–12 months postpartum in Nepal. Consistent with recommendations of prior studies (Draucker et al. 2020; Fetters et al. 2013), the joint display and narrative weaving approaches were used for presenting both quantitative and qualitative findings (Table 4). Notably, the mixed‐method findings revealed a lack of spousal and social support and IPV as important contributing factors to depression in women during the 6–12 months postpartum period. This aligns with a systematic review of 589 studies across 51 countries, which found the highest PPD prevalence (38.9%) among women who experienced IPV during pregnancy and up to 12 months postpartum (Roddy Mitchell et al. 2023). A meta‐analysis of 12 studies further reported that verbal and physical abuse significantly increased the odds of PPD (aOR = 2.89 and 2.29, *p* = 0.001 and 0.002, respectively) (Putri et al. 2023). Another meta‐analysis found stronger associations between IPV and PPD in low‐ and middle‐income countries compared to high‐income settings (Wang et al. 2021). Our findings are also consistent with other prior studies, indicating that IPV is a significant risk factor for PPD (Abdoli Najmi and Mirghafourvand 2024; Dadi et al. 2020; Mulyani et al. 2023). Other prior studies also indicated IPV as a significant risk factor for PPD (Abdoli Najmi and Mirghafourvand 2024; Dadi et al. 2020; Mulyani et al. 2023). Some participants in this study also reported self‐harming behaviours and violence tendencies, which is similar to recent study results among a Nepali sample (Neupane et al. 2024).

Similar to this study's findings, prior evidence from Nepal and Ghana reported women experiencing a range of negative emotions such as fear, anxiety, frustration, sadness, worthlessness, hopelessness and low self‐esteem during postpartum (Dartey et al. 2024; Holopainen and Hakulinen 2019; Neupane et al. 2024; Subba et al. 2024). In a systematic review by Holopainen and Hakulinen (2019), women expressed feelings of anger, despair, hopelessness and helplessness due to inadequate support from their spouses, families, and significant others and financial worries. Consistent with findings of a prior study in Nepali sample (Subba et al. 2024), postpartum women in this study also reported having various physical symptoms, including headaches, bodyaches, tachycardia, chest heaviness and difficulty breathing requiring frequent medical attention for these complaints. Although a referral for consultation with a psychiatrist were recommended for women with higher depressive symptoms and increased mental, emotional and somatic complaints, many women declined these referrals—likely due to a lack of awareness of mental health services and the stigma associated with mental health care in Nepal. Crying was identified as one of the major coping mechanisms used by participants in our study which aligns with findings from an earlier study among Nepali sample (Subba et al. 2024). Additionally, a study in Ghana reported that some women adopted self‐medication and seeking friends or family support as coping strategies for PPD (Dartey et al. 2024).

A meta‐analysis of 18 cross‐sectional studies (2013–2023) found that high family income significantly reduced the risk of PPD (aOR = 0.50; 95% CI: 0.28–0.90; *p* = 0.020), identifying low income as a major contributing factor (Mulyani et al. 2023). Consistent with a prior systematic review of 86 studies across various countries (Pan et al. 2024), our mixed‐method findings highlight strong social support, particularly daily family involvement in childcare, as a protective factor against PPD. For instance, spouse support and strong family engagement further contribute to reducing PPD symptoms (Wasti et al. 2024). In many cultural contexts, including Nepal, family support during the early postpartum period is both expected and essential for women's physical and emotional well‐being.

Infant care during the postpartum period is particularly challenging for women, especially first‐time mothers. Consistent with previous findings (Coates et al. 2014; Neupane et al. 2024), in this study, qualitative participants reported experiencing negative emotions and difficulty adjusting to their new role—such as difficulty with breastfeeding—that sometimes led to inadequate bonding with their child. These challenges were more common among mothers of LBW/preterm or sick infants (Neupane et al. 2024), highlighting the need for additional emotional and practical support during the postpartum period for at‐risk women (Coates et al. 2014). Other studies have also identified that infant health‐related complications and LBW/preterm birth as additional risk factors for PPD (Amer et al. 2024; Pudasainee‐Kapri et al. 2025; Wang et al. 2021). Interestingly, women with preterm and low birth weight infants having adequate spouse and family support expressed less stress and good adjustment indicating social support as an important factor. While families focus primarily on the newborn, the mothers' physical and emotional needs are frequently overlooked. Additionally, many women reported feeling unprepared to take care of their infant and handle postpartum challenges, contributing to psychological distress (Neupane et al. 2024). Another study highlighted higher levels of education as protective factors for PPD (Pan et al. 2024). Consistent with findings from prior studies (Dadi et al. 2020; Pan et al. 2024), this study also identified that anxious and depressive thoughts during pregnancy, low family income, partner substance use, male‐child preference, and food insecurity as major risk factors for PPD.

Similarly, a cross‐sectional study among 300 women at a hospital in Kathmandu, Nepal, identified several risk factors for increased prevalence of perinatal depression including unsupportive family members, delivery complications, history of IPV and first pregnancy at age ≤ 25 years (Wasti et al. 2024). Women who participated in the qualitative study also reported emotional distress stemming from difficulty adapting to a different culture or social context of their spouse's home due to inter‐caste marriage, restricted personal freedom, limited decision‐making power and a lack of partner and/or family support. Unsatisfying marital relationships further contributed to feelings of loneliness, sadness and hopelessness (Subba et al. 2024). Likewise, financial constraints or being dependent on their spouse, the burden of household work, unplanned pregnancies, cultural preference for a son, and past traumatic experiences were some of the leading factors for depressive symptoms among postpartum women.

In particular, the findings of this study are also supported by a recent study among 173 postpartum women attending an immunisation clinic in Nepal in which having a female child, unplanned pregnancy, and childbirth‐related health problems were significantly associated with PPD (Bhusal et al. 2023). A prior study of postpartum women 1–6 months postpartum also identified low birth weight and lack of social support as significant factors associated with PPD (Pudasainee‐Kapri et al. 2025). Consistent with these findings, Subba et al. (2024) emphasised that a lack of family support, cultural pressure and partner neglect frequently exacerbated emotional struggles among postpartum women. Although husbands were often seen as a major source of strength, the absence of their support, coupled with poor family dynamics, left many women feeling isolated and hopeless. Prior research described peer support as an accessible, effective and non‐stigmatising form of emotional assistance for postpartum mothers and played a key role in enhancing women's ability to seek and utilise mental health services (Rice et al. 2022). Participants in this study also perceived that peer support and family support were protective factors against their poor postpartum mental health.

### 4.1 Limitations

Although this study offers valuable insights on depression during a later postpartum year, there are some notable limitations. First, the use of self‐reported data on depressive symptoms introduces the potential for recall bias. Similarly, stigma related to mental health issues may impact participants' responses. Likewise, self‐reported data on IPV has the potential for social desirability bias and stigma rooted in patriarchal norms. Second, this study cannot establish causal relationships due to its cross‐sectional design; longitudinal studies with data collected at multiple time points are needed. Third, the study was conducted at the immunisation clinics of two hospitals in Kathmandu using convenience sampling, thereby limiting the generalizability of the findings to all postpartum women in other geographic regions or at the national level.

## 5 Conclusion and Implications

To our knowledge, this is the first mixed‐method study that focused on depression among women 6–12 months postpartum in Nepal. Findings from this study highlight the importance of addressing depression among women not only during the immediate postpartum period but throughout the first year following childbirth. Results of this study have important implications to improve maternal mental health, promote healthy families and communities, and influence policymaking regarding practices around perinatal mental health. Most notably, nurses, midwives, obstetric and paediatric providers should conduct routine screening using well‐validated instruments to identify women at‐risk for depression and ensure timely treatment and referral during pregnancy and throughout the first year postpartum. Findings can be used to implement actionable practice change for routine screening at immunisation clinics, strengthening partner and family support throughout the first postpartum year, and enhancing referral pathways for women experiencing depression and family/partner violence. Additionally, societal awareness campaigns are needed to reduce the stigma surrounding mental health issues in Nepal. Culturally tailored, community‐based interventions are also essential in improving maternal mental health, promoting health equity, and enhancing maternal and infant outcomes. These efforts align with Nepal's Sustainable Development Goals (SDGs) to improve maternal and child health outcomes. Emerged from this study is the need for additional research examining mechanisms and longitudinal pathways surrounding perinatal depression among women.

## Funding

This research was made possible in part with support from a Global Health Seed Grant from Rutgers Global Health Institute and Rutgers Global at Rutgers, The State University of New Jersey.

## Ethics Statement

This study was approved by the Institutional Research Board of Rutgers University (# Pro2023000179), the Ethical Review Board of the Nepal Health Research Council (#45), and the Institutional Research Committees (IRCs) of the two research sites. After obtaining those approvals, participant written informed consent was obtained prior to data collection. Privacy and confidentiality of participants were maintained throughout the study and during dissemination of findings. Surveys, interview recordings and any notes taken during the interviews were securely stored in a locked file cabinet and on a password‐protected laptop. The transcripts were kept confidential, and precautions were taken to protect participants' personal information.

## Conflicts of Interest

The authors declare no conflicts of interest.

## Data Availability

The data that support the findings of this study are available on request from the corresponding author. The data are not publicly available due to privacy or ethical restrictions.
